# Fire up

**Published:** 1857-02

**Authors:** 


### "FIRE UP,"

Is the watchword, when the steamer is ready for motion. "Fire up" is the order when the "Iron Horse" is going to ride us on a rail; and just as necessary is it for a man to "Fire up," if he means to accomplish anything; but the fuel for his firing is not exactly wood and water; his furnace is not exactly made of iron, but he must be fed and watered every day, or a stagnation would prevail in every department of human life.

Very close calculations have been made to ascertain how much it takes to feed a steamship or locomotive. *Wall street* is very specially interested in the decision of that question. A locomotive eats up a cord of wood in an hour, while a steamship consumes eighty tons of coal in a single day; but to keep the machine of man in motion for a day, or month, or year, how interesting a question!—we mean, thereby, the hardy, industrious farmer or mechanic, for they have the first right to a "*liberal allowance;*" but if the lazy, the lounging, the do-nothing nobodies, who encumber our earth, appropriate to themselves this "liberal allowance," they are fined in a sum of ten or twenty years of existence, *and that fine must be paid*, more irrevocable than any Medo-Persian edict. A man, then, who works hard out of doors, every day, will, in a year, consume

Eight hundred pounds of air,
Eight hundred pounds of food,
Fifteen hundred pounds of water.

In other words, it requires three thousand and two hundred pounds of air, food and water, to sustain a man for one year; but if he be a gentleman loafer, such as occupy *our* poor-houses, hospitals and asylums, about seven hundred pounds of all kinds of food are required.

The practical use which we wish to make of these statements is, if eight hundred pounds of meat and bread and vegetables go down our throats every year, are every year introduced into our bodies, it is just as necessary that the refuse should be passed out of them, with regularity, as it is for the ashes to be removed from the furnace of the steamship or locomotive. If a single day's ashes and cinders are unremoved, the finest machine in the world would cease to run. Just as inevitable is the derangement of the whole machinery of man, if, for a single day, he does not pass from his body an amount equal to what he ate the day before. And one of the most important items we have ever given in the pages of this journal is: *whenever the hour of bowel action has passed by, without its occurrence, do not swallow an atom of food until it takes place.* This alone would remove the cause of half our diseases.

